# Bonfire red titles

**DOI:** 10.1007/s40037-016-0267-3

**Published:** 2016-05-23

**Authors:** Lorelei Lingard

**Affiliations:** Schulich School of Medicine & Dentistry, Health Sciences Addition, Western University, London, Ontario Canada

In the writer’s craft section we offer simple tips to improve your writing in one of three areas: Energy, Clarity and Persuasiveness. Each entry focuses on a key writing feature or strategy, illustrates how it commonly goes wrong, teaches the grammatical underpinnings necessary to understand it and offers suggestions to wield it effectively. We encourage readers to share comments on or suggestions for this section on Twitter, using the hashtag: #how’syourwriting? See previous such articles.

A few years ago I painted my front door red. The rest of my house was fairly conservative: beige wood siding and white trim on a classic, two-story design. To the rest of my street, it said, “I am like you; I belong here”. Of course, it also said, “Nothing particularly exciting is going on inside these walls.” So one morning I biked to the hardware store, returned with a can of “Bonfire Red” paint and fixed that little bit of false advertising.

A title is like a front door: it serves as advertising for what’s inside your research paper. Have a look at the last title you wrote for an academic manuscript. Is it a red door or a white one? Does it draw readers into your work or encourage them to walk by?

Titles must achieve two goals: quickly grab the reader and faithfully describe the paper. This likely explains our common addiction to the “colon title”, in which what precedes the colon is meant to be catchy and what follows is meant to be descriptive [1]. Sometimes, this structure is effective:

Building the ark: A faculty development strategy for surviving the assessment demands of competency-based medical education.

This title has a visual impact: “the ark” and “surviving” call up the biblical story. And it uses this metaphor to imply a stance in the controversial global shift to competency-based medical education – a flood of assessment is coming to drown us. In qualitative papers, an iconic quote can create an effective variant of the “colon title”:

*“You learn better under the gun”: Intimidation and harassment in surgical education *[2]*.*

By leading with the voice of a study participant, this title brings the reader into the world of surgical training.

But the “colon title” can just as easily let us down. The structure often lures us to write titles like this one:

The implications of competence-by-design for the next generation of faculty development strategies: An online Delphi survey of leaders in Canadian medical education.

In contrast to the earlier examples, this title is weighed down by an abundance of abstractions (“implications”, “generation”, “medical education”) and jargon (“competence-by-design”, “online Delphi survey”).

Specialized jargon abounds in academic titles, suggesting that many writers believe it is necessary, perhaps to signal their membership in an expert research community. It is not necessary, as the title of this highly cited paper illustrates:

*What every teacher needs to know about clinical reasoning* [3]*.*

There is no jargon in this title. Its claim that the paper includes a message about “what every teacher needs to know” is reflected in its candid and accessible language.

While many of us recognize that catchy is better than boring, we’ve all seen examples where a catchy title failed. For one thing, it can be a slippery slope from catchy to cutesy:

This little piggy …: Ten tips for teaching novices about the healthcare marketplace.

And perhaps, like me, you’ve had editors ask you to change a catchy title to something more conventional: “*Your title does not closely enough reflect the work described: please revise to more accurately depict the study’s method and results.” *Such requests are a reminder that “catchy” and “nuanced” are difficult to achieve simultaneously, and the writer must balance them artfully.

How do you know when a catchy title will work, and when it will not? Sword’s advice is to attend not only to the title’s text (what is said), but also to its paratext and subtext (what is implied). All three contribute to its impact and effectiveness in a given context [1]. Paratext is any extra-textual matter that accompanies and influences the meaning of a title. In a journal, this includes author names and affiliations, journal name, perhaps the topic of a special issue. Consider this example:

*RCT = results confounded and trivial: The perils of grand educational experiments *[4]*.*

The author of this article is a senior researcher in the medical education field, which adds paratextual meaning to this title. It says, “this title’s stance may be playful but the paper is written by a respected researcher and therefore should not be taken lightly”. This paratextual dimension may explain why some researchers are reluctant to craft more daring titles; if they do not feel confident in their established ethos in the field, they may feel that work titled playfully could be dismissed as frivolous.

Subtext is any message in the title that is not stated explicitly in words but can be inferred. In the “Building an ark” example, the “ark” reference tells the reader “I will try to entertain you, and I won’t back away from controversy”. The deliciously sacrilegious translation of RCT as “results confounded and trivial” rather than “randomized controlled trial” implies the subtext, “I am sufficiently confident to oppose a dominant scientific assumption”. Whether either paper will bear out these subtextual messages is another matter, but they are part of what the title advertises to the reader.

Attention to paratext and subtext can help writers be more strategic in the titles they create. Ask yourself, what does the journal context imply? For instance, if you’re publishing in *Qualitative Health Research*, then using the phrase “qualitative research study” in your title is likely redundant. Similarly, a colon title that is provocative on one side and conventional on the other can subtextually imply that the work is balanced – both catchy and solid:

Secrets, lies and manipulation: Exploring how family health teams negotiate authority.

Remember, though, that in this example “secrets, lies and manipulation” must feature strongly in the study results, or this title will be guilty of setting up expectations that the paper cannot satisfy.

Two-part colon titles are dominant in research writing, but they are not necessary. For many writers, they have simply become automatic. The next time you write a colon title, critically analyze it: do you really need both sections? Consider this example:

Is saturation really possible?: A content analysis of saturation claims in qualitative medical education research.

A single clause could achieve the same meaning, with a more punchy tone:

The impossibility of saturation.

While this version does not reference the study methodology (content analysis) or the study setting (medical education), there is no consistent rule that titles must reference either. If the methodology is in the paper’s keywords, it will be searchable on that basis. If this is a medical education journal, the study setting will be implied paratextually. As a qualitative medical education researcher, I’d not only read a paper about *The impossibility of saturation* – I’d read it right away!

When journals offer specific guidelines for titles, writers should try to abide by them. This journal, for instance, suggests that the title include “all information … that will make electronic retrieval of the article both sensitive and specific”; it also, however, acknowledges that “concise titles are easier to read than long, convoluted ones” [5]. The best way to know if you’re balancing the dimensions of a good title is to make a short list of titles and ask readers for feedback: Does the title grab them? Does the paper follow through? And, as a general rule, if you can have a bonfire red door rather than a white one, paint it!

## References

[1] Sword H (2012). Stylish Academic Writing.

[2] Musselman L, Reznick R, MacRae H, Lingard L (2005). You learn better under the gun: Intimidation and harassment in surgical education. Med Educ.

[3] Eva K (2005). What every teacher needs to know about clinical reasoning. Med Educ.

[4] Norman G (2003). RCT = results confounded and trivial: the perils of grand educational experiments. Med Educ.

[5] Instructions for Authors. Perspectives on Medical Education. http://www.springer.com/education+%26+language/journal/40037. Accessed March 9, 2016.

